# Fc Gamma Receptors and Complement Component 3 Facilitate Anti-fVIII Antibody Formation

**DOI:** 10.3389/fimmu.2020.00905

**Published:** 2020-06-09

**Authors:** Patricia E. Zerra, Connie M. Arthur, Satheesh Chonat, Cheryl L. Maier, Amanda Mener, Sooncheon Shin, Jerry William L. Allen, W. Hunter Baldwin, Courtney Cox, Hans Verkerke, Ryan P. Jajosky, Christopher A. Tormey, Shannon L. Meeks, Sean R. Stowell

**Affiliations:** ^1^Department of Pathology and Laboratory Medicine, Center for Transfusion Medicine and Cellular Therapies, Emory University School of Medicine, Atlanta, GA, United States; ^2^Aflac Cancer and Blood Disorders Center at Children's Healthcare of Atlanta and Department of Pediatrics, Emory University School of Medicine, Atlanta, GA, United States; ^3^Department of Laboratory Medicine, Yale University School of Medicine, New Haven, CT, United States; ^4^Pathology and Laboratory Medicine Service, VA Conneciticut Healthcare System, West Haven, CT, United States

**Keywords:** hemophilia, inhibitors, Fc gamma receptors, complement component 3, alloimmunization, humoral immunity

## Abstract

Anti-factor VIII (fVIII) alloantibodies, which can develop in patients with hemophilia A, limit the therapeutic options and increase morbidity and mortality of these patients. However, the factors that influence anti-fVIII antibody development remain incompletely understood. Recent studies suggest that Fc gamma receptors (FcγRs) may facilitate recognition and uptake of fVIII by recently developed or pre-existing naturally occurring anti-fVIII antibodies, providing a mechanism whereby the immune system may recognize fVIII following infusion. However, the role of FcγRs in anti-fVIII antibody formation remains unknown. In order to define the influence of FcγRs on the development of anti-fVIII antibodies, fVIII was injected into WT or FcγR knockout recipients, followed by evaluation of anti-fVIII antibodies. Anti-fVIII antibodies were readily observed following fVIII injection into FcγR knockouts, with similar anti-fVIII antibody levels occurring in FcγR knockouts as detected in WT mice injected in parallel. As antibodies can also fix complement, providing a potential mechanism whereby anti-fVIII antibodies may influence anti-fVIII antibody formation independent of FcγRs, fVIII was also injected into complement component 3 (C3) knockout recipients in parallel. Similar to FcγR knockouts, C3 knockout recipients developed a robust response to fVIII, which was likewise similar to that observed in WT recipients. As FcγRs or C3 may compensate for each other in recipients only deficient in FcγRs or C3 alone, we generated mice deficient in both FcγRs and C3 to test for potential antibody effector redundancy in anti-fVIII antibody formation. Infusion of fVIII into FcγRs and C3 (FcγR × C3) double knockouts likewise induced anti-fVIII antibodies. However, unlike individual knockouts, anti-fVIII antibodies in FcγRs × C3 knockouts were initially lower than WT recipients, although anti-fVIII antibodies increased to WT levels following additional fVIII exposure. In contrast, infusion of RBCs expressing distinct alloantigens into FcγRs, C3 or FcγR × C3 knockout recipients either failed to change anti-RBC levels when compared to WT recipients or actually increased antibody responses, depending on the target antigen. Taken together, these results suggest FcγRs and C3 can differentially impact antibody formation following exposure to distinct alloantigens and that FcγRs and C3 work in concert to facilitate early anti-fVIII antibody formation.

## Introduction

Undetectable levels of circulating factor VIII (fVIII) in most patients with severe hemophilia A not only results in impaired coagulation, but also fails to induce immunological tolerance to fVIII during neonatal and early life (1, 2). As a result, therapeutic exposure to exogenous fVIII can induce the formation of inhibitory anti-fVIII antibodies (inhibitors), which render fVIII therapy ineffective (3–9). This, in turn, makes bleeding difficult to control and prevent, resulting in increased morbidity and mortality, increased cost of care and decreased quality of life (5, 8). fVIII inhibitors occur in ~20–30% of patients with severe hemophilia A and 5% of patients with mild/moderate hemophilia A, and represent one of the most significant complications in the management of patients with hemophilia A (3–12).

One of the most common approaches to inhibitor eradication is immune tolerance therapy (ITT). However, while ITT is successful in 60–70% of cases, this treatment continues to suffer from the significant time and expense required for implementation (8, 13–16). In addition, while the relatively new chimeric antibody, emicizumab, can provide effective prophylaxis to reduce bleeding risk in patients with inhibitors, it does not treat acute bleeding events (17–19). As such, patients with inhibitors continue to be difficult to manage during acute bleeding episodes (e.g., trauma, surgery, etc.).

Despite the negative consequences of inhibitor formation, no prophylactic therapy is currently available to prevent inhibitor development. This in part reflects a fundamental lack of understanding regarding the key immune regulators that govern inhibitor formation. Recent studies suggest that several key initiating immune cells, including marginal zone macrophages (MZM) and marginal zone (MZ) B cells, may be responsible for initiating inhibitor development (20, 21). However, while these and other cells may influence inhibitor formation (22–29), current paradigms in immunology suggest that a “danger signal” must be present to appropriately activate immune cells and therefore drive adaptive immune responses toward foreign antigens (30–37). As fVIII is an otherwise innocuous antigen, the innate immune stimuli responsible for triggering fVIII immune responses has remained unknown (38–40). Given the challenges associated with optimally managing hemophilia A patients with inhibitors (5, 8), a greater understanding of key factors that influence inhibitor development is needed.

Previous studies suggest that early antibody formation or pre-existing naturally occurring anti-fVIII antibodies may engage fVIII (41–43), thereby facilitating additional anti-fVIII antibodies following subsequent exposure. As antibody ligation of dendritic cells, macrophages and other immune cells can lead to immune cell activation (44), anti-fVIII antibodies could provide the innate immune signaling events required for activation of the adaptive immune system, while also enhancing the detection and uptake of fVIII by key immune populations responsible for orchestrating a productive immune response (45). Consistent with this, incubation of anti-fVIII antibodies with fVIII can enhance fVIII uptake *in vitro*, while injection of antibody-fVIII complexes *in vivo* can enhance *de novo* anti-fVIII antibody formation (41–43). Taken together, these results suggest that antibody engagement and trafficking of fVIII to appropriate immune cells may enhance anti-fVIII antibody formation.

While several studies suggest that antibody engagement can enhance anti-fVIII antibody development, whether anti-fVIII antibodies that develop in response to fVIII likewise regulate an ongoing fVIII immune response remains unknown. Enhancement of *de novo* inhibitor development by existing anti-fVIII antibodies is thought to occur primarily through Fcγ receptor (FcγR) engagement of antibody-fVIII complexes (41, 42, 45), resulting in the endocytosis, activation and presentation of fVIII to key components of the immune system. In this way, antibody engagement of fVIII may enhance fVIII removal, while also targeting fVIII to appropriate immune populations capable of facilitating an overall fVIII immune response. However, while interactions between affinity matured anti-fVIII antibodies and fVIII appear to enhance fVIII immunogenicity, the actual role of FcγRs on the developing anti-fVIII immune response remains unknown.

## Materials and Methods

### Mice and Materials

Female C57BL/6 (B6) recipients were purchased from the National Cancer Institute (Frederick, MD) or Charles River (Wilmington, MA) and used as wild-type (WT) controls for each experiment. C3 knockout (B6;129S4-C3^tm1Crr^/J) and FcγR knockout (B6;129P2-Fcer1g^tm1Rav^/J) mice were purchased from Jackson Laboratories (Bar Harbor, ME) and Taconic Biosciences (Renesselaer, NY), respectively. Recipients deficient in C3 and Fcγ receptors (FcγR x C3 knockouts) were generated as outlined previously (46). Transgenic KEL and HOD donors were maintained as outlined previously (47, 48). fVIII knockout mice (hemophilia A mice, TKO) on a C57BL/6 background were used for complement depletion experiments; these mice possess a deletion of the entire *F8* coding sequence (40). A combination of male and female mice, all aged 8 to 12-weeks-old were used. All animals were housed and bred in cages at the Emory University Department of Animal Resources facilities, and all experiments were performed under animal protocols approved by the Institutional Animal Care and Use Committee of Emory University. Full-length recombinant human fVIII (rfVIII) was generously donated by Hemophilia of Georgia and Christopher Tormey, Yale University. Native cobra venom factor (nCVF) from *Naja naja kaouthia* was used for complement depletion studies (Quidel Corporation, Athens, OH).

### fVIII Immunization Regimen

B6, FcγR knockout, C3 knockout, FcγR x C3 knockout and hemophilia A mice received human full-length rfVIII in a 100-μL total volume of sterile saline via retro-orbital injection. fVIII was administered according to previously described dosing and administration schedules (21, 40). Briefly, mice received weekly doses of 2 μg fVIII for 2–4 weeks. In B6 or FcγR x C3 knockout mice receiving a “boost” dose, a 4 μg fVIII dose was given 1 week after the 4th dose as outlined previously (21, 40). Hemophilia A mice were administered fVIII 6 h after receiving 7.5 U nCVF via intra-peritoneal injection.

### Plasma Analysis for Anti-fVIII Antibodies

To examine anti-fVIII antibody formation in B6, FcγR knockout, C3 knockout, FcγR × C3 knockout or hemophilia A mice, blood was collected from the orbital venous plexus with heparinized capillary tubes into 3.8% sodium citrate at 1:10 dilution 7 days after the last injection of fVIII for all specified time points. Samples were then microcentrifuged at 3,200 rpm for 15 min, with resulting plasma collected and frozen until further analysis. To measure anti-fVIII IgG titers, an enzyme-linked immunosorbent assay (ELISA) was performed, as previously described (21, 39, 49).

### Characterization of Mice: C3 Levels and Fcγ Receptors

To examine C3 protein levels in serum from B6, FcγR knockout, C3 knockout, FcγR x C3 knockout or hemophilia A mice, an ELISA was performed using a mouse C3 ELISA Kit from Abcam (Cambridge, MA). To verify the presence or absence of Fc**γ** receptors in B6, C3 knockout, Fc**γ**R knockout and FcγR x C3 knockout mice, peripheral blood was collected via tail vein into ACD, followed by red blood cell lysis with Ammonium-Chloride-Potassium Lysing Buffer (ThermoFisher). Lymphocytes were then stained with V500 anti-CD45R/B220, PerCP Cy5.5 anti-CD11b, PE anti-CD11c and allophycocyanin anti-CD16 (BioLegend, San Diego, CA) diluted in fluorescence-activated cell sorting (FACS) buffer (PBS + 2% BSA) for 30 min at 4°C. The mean fluorescent intensity of FcγRI (CD16) present on CD11b positive peripheral blood leukocytes in each mouse was determined using an LSR-II flow cytometer (BD Biosciences) and analyzed using FlowJo software version 10.4.2.

### Red Blood Cell (RBC) Isolation and Staining

HOD or KEL RBCs were collected into a 50 mL conical tube containing 1:8 ACD as outlined previously (47, 48, 50, 51). For incompatible transfusion experiments, HOD or KEL RBCs were labeled with Molecular Probes Cell Tracker CM-DiI, (1,1'-dioctadecyl-3,3,3'3'-tetramethylindocarbocyanine perchlorate). Control B6 blood was labeled with another lipophilic dye, DiO (3,3'-dihexadecyloxacarbocyanine perchlorate), as previously described (47, 52). DiI and DiO labeling was confirmed individually by flow cytometry prior to mixing and transfusion. DiI KEL RBCs or DiI HOD RBCs were mixed with DiO B6 RBCs equally. Each mouse was transfused with 50 μL DiI KEL RBCs or DiI HOD RBCs (1:1 with DiO B6 RBCs) resuspended in 300 μL PBS into the lateral tail vein. For alloimmunization experiments, HOD or KEL RBCs were similarly collected and 50 μL of unlabeled packed HOD or KEL RBCs were transfused into each recipient (47, 48, 50, 51).

### Peripheral Blood Staining

Following transfusion, peripheral blood was collected by retro-orbital bleeding of each mouse into ACD and washed 3x in FACS buffer. Peripheral blood was then stained for the HOD or KEL antigen using anti-KEL or anti-HOD antibodies, respectively, in FACS buffer as outlined previously (47, 48, 50, 51). Stained RBCs were then washed 3x in FACS buffer, followed by incubation with a secondary antibody, anti-mouse IgG APC (Jackson Immunoresearch) in FACS buffer, for 20 min at room temperature. Stained RBCs were then washed 3x in FACS buffer and diluted to a final volume of 100 μL in FACS buffer. Complement was detected through biotinylated antibodies against mouse C3 (Cedarlane) followed by streptavidin APC (BD). 50 μL of each set of stained RBCs in FACS buffer was added to 400 μL of FACS buffer and the level of complement was measured by a FACSCalibur flow cytometer (47, 52).

### Serum Analysis for Anti-RBC Antibodies

The presence of anti-KEL and anti-HOD antibodies was evaluated through indirect immunofluorescent staining of serum collected from transfused recipients on day 14 after RBC transfusion, as described previously (51–53). Briefly, serum was combined with packed KEL, HOD or B6 RBCs for 15 min at room temperature. After washing with FACS buffer, samples were incubated with APC anti-mouse IgG for 30 min. The amount of antigen specific antibody present in each sample was measured by subtracting the signal obtained following serum incubation with B6 RBCs alone from the signal observed following similar incubation with HOD or KEL RBCs, respectively. Flow cytometric data was acquired using CellQuest Pro and analyzed using FlowJo software version 10.4.2.

### Statistical Analysis

Unpaired *t*-test or one-way analysis of variance (ANOVA) test with a *post hoc* Tukey's multiple comparisons test were performed to determine significance of results. Prism 8.2 (GraphPad Software, La Jolla, CA) was used to perform all statistical analyses. *P*-values < 0.05 were considered statistically significant.

## Results

### Anti-fVIII Antibodies Can Form Independent of FcγRs or C3

**Figure 1:**
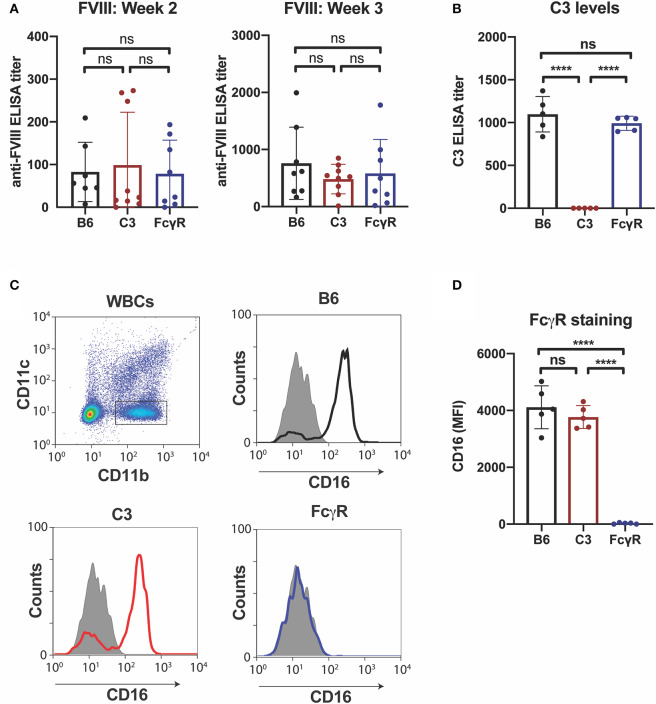
Anti-fVIII antibodies can form independent of Fcγ receptors or C3. **(A)** WT, C3 knockout or Fcγ receptor knockout recipients received 3 weekly injections of fVIII followed by evalution of anti-fVIII antibody formation by ELISA. **(B)** Analysis of C3 levels in WT, C3 knockout and FcγR knockout mice. **(C)** Flow cytometry gating strategy used to examine Fcγ R1 (CD16) expression on the surface of leukocytes. **(D)** Quantiative analysis of Fcγ receptor levels in WT, C3 knockout or Fcγ receptor knockout recipients. ns = not significant. ****p < 0.0001.

Given the possible role of FcγRs in the developing immune response to fVIII, we first sought to define the role of FcγRs by leveraging mice completely deficient in the common γ chain used by all activating FcγRs (FcγRs I, III, and IV), a common approach to examine FcγR function (44). As recent data also demonstrate that C3 can regulate anti-fVIII antibody formation (27), and antibody engagement of antigen can also induce C3 activation (54), we also examined the role of C3 in anti-fVIII antibody formation by using C3 knockout mice, which are genetically deficient in C3, in parallel. To accomplish this, we injected rfVIII (2 μg) weekly into either WT, FcγR knockout or C3 knockout recipients. To examine the potential influence of these immune factors early in the development of anti-fVIII antibodies, plasma was harvested 21 days following initial fVIII injection and evaluated for anti-fVIII antibodies. Unexpectedly, anti-fVIII antibodies readily formed in FcγRs knockout recipients following fVIII exposure (Figure 1A). Similarly, C3 knockouts were also responsive to fVIII infusion (Figure 1A). Indeed, the development of anti-fVIII antibodies between WT, FcγR knockouts and C3 knockouts was not statistically different, with similar antibody responses being observed 21 days post initial fVIII exposure (Figure 1A).

**Figure 2:**
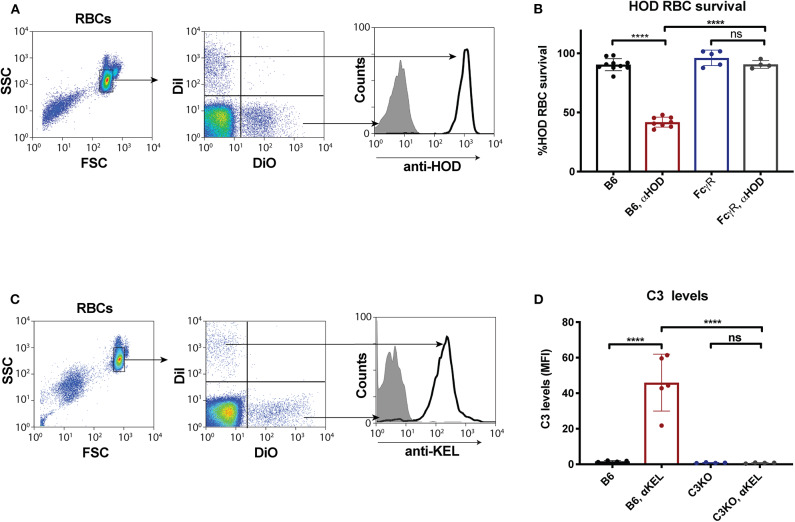
Fcγ receptor and C3 knockouts exhibit an impaired ability to mediate RBC clearance or C3 deposition following incompatible RBC transfusion. **(A)** HOD (HEL, OVA, and Duffy) RBCs labeled with the lipophilic dye, DiI, can be discriminated from WT B6 RBCs labeled with a distinct lipophilic dye, DiO, following transfusion into a WT recipient. **(B)** HOD RBCs were transfused into non-immunized or anti-HOD (αHOD) immunized WT or Fcγ receptor knockout recipients, followed by evaluation for specific HOD RBC clearance. **(C)** KEL RBCs labeled with the lipophilic dye, DiI, can be discriminated from WT B6 RBCs labeled with a distinct lipophilic dye, DiO, following transfusion into a WT recipient. **(D)** KEL RBCs were transfused into non-immunized or anti-KEL (αKEL) immunized WT or C3 knockout recipients, followed by examination for C3 deposition specifically on the KEL RBC surface. ns = not significant. ****p < 0.0001.

Given the unexpected outcome of anti-fVIII antibody formation observed in FcγRs and C3 knockout recipients, it remained possible that residual FcγRs or C3 may be present in these recipients. To initially test this, we analyzed C3 levels in WT, FcγR knockout and C3 knockout recipients. While C3 was variable, yet present, in WT and FcγR knockout recipients, we failed to detect C3 in C3 knockout recipients (Figure 1B). Similarly, to confirm that FcγRs were absent in FcγR knockout recipients, we examined peripheral blood leukocytes for CD16 expression and found that while leukocytes harvested from WT and C3 knockout mice readily expressed CD16, this FcγR was completely absent in FcγR knockouts (Figures 1C,D). However, given the unexpected outcome of fVIII infusion in these recipients, to firmly establish whether residual FcγR or C3 function may be present, we utilized two incompatible RBC transfusion models shown to be entirely dependent on FcγRs or to result in detectable C3 fixation on the cell surface, respectively (47, 52). To accomplish this, HOD RBCs, which express the HOD antigen (a chimeric fusion protein of HEL, OVA and Duffy) were labeled with a lipophilic dye, DiI, to facilitate detection post-transfusion and mixed with HOD antigen negative RBCs labeled with a fluorescently distinct dye, DiO. While transfusion of HOD RBCs into immunized recipients resulted in robust clearance, no detectable HOD RBC removal was observed following transfusion of HOD RBCs into immunized or non-immunized FcγR knockouts (Figures 2A,B). Antibody binding to HOD RBCs does not fix appreciable complement (47). As a result, we next examined C3 deposition following transfusion of RBCs expressing the KEL antigen using a similar experimental approach. Incompatible KEL RBC transfusion resulted in significant C3 deposition, while similar transfusion into C3 knockout mice failed to result in detectable C3 on the KEL RBC surface (Figures 2C,D). Taken together, these results demonstrate that FcγR and C3 knockouts are deficient in FcγRs and C3 activity, respectively, and that anti-fVIII antibody formation, therefore, does not appear to require FcγRs or C3 in this model system.

### Alternative Intravascular Antigens Induce Antibodies Independent of FcγRs or C3

**Figure 3:**
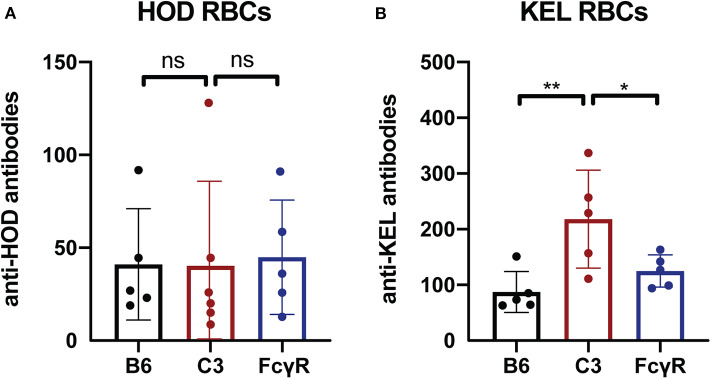
C3 has a differential impact on anti-RBC antibody formation depending on the target antigen. **(A)** Flow cross match results obtained following transfusion of HOD RBCs into WT B6, C3 knockout or Fcγ receptor knockout recipients. **(B)** Flow cross match results obtained following transfusion of KEL RBCs into WT B6, C3 knockout or Fcγ receptor knockout recipients. ns = not significant. * = < 0.04, ** = < 0.009.

To determine whether the immune response to other intravascular antigens likewise occurs in the absence of FcγRs, as a control, we next examined the outcome of transfusing RBCs expressing the same alloantigens used to define FcγR or C3 activity; like fVIII, these antigens are delivered intravascularly. To examine this, FcγR knockout recipients were transfused with either HOD or KEL RBCs, followed by examination of anti-HOD or anti-KEL antibody formation, respectively. Similar to the development of anti-fVIII antibodies, HOD RBCs and KEL RBCs were able to induce anti-HOD and anti-KEL antibodies irrespective of the presence or absence of FcγRs (Figures 3A,B). As an additional control, HOD or KEL RBCs were likewise transfused into C3 knockout recipients in parallel. Similar to transfusion into FcγR knockout recipients, HOD or KEL RBC transfusion into C3 knockout recipients resulted in robust anti-HOD and anti-KEL antibody formation, with anti-KEL antibodies formation in C3 knockout recipients actually displaying an enhanced response when compared to WT recipients (Figures 3A,B). These results demonstrate that like fVIII, HOD and KEL RBCs appear to possess the ability to induce antibody formation in absence of functional FcγRs or C3.

### Examination of Complement Depletion on Early Anti-fVIII Antibody Formation

As C3 knockout mice have normal levels of mouse fVIII, we next aimed to investigate the role of complement in FVIII-deficient hemophilia A mice. To accomplish this, we depleted complement by administering nCVF to WT or hemophilia A mice. Plasma C3 levels were determined in mice at baseline and 6, 12, and 24 h after nCVF injection, which showed C3 depletion by 6 h that persisted for 24 h (Supplementary Figure 1A). Next, two weekly doses of saline or 7.5 U nCVF were administered to WT or hemophilia A mice followed by rfVIII 6 h later (Supplementary Figure 1B). Plasma C3 levels were obtained 24 h after each nCVF administration to ensure adequate complement depletion was attained (Supplementary Figure 1C). Plasma collected 14 days following initial fVIII injection was analyzed for anti-fVIII antibody formation with ELISA. Similar to C3 knockout mice, B6 or hemophilia A mice that received nCVF prior to fVIII injection produced inhibitors at the same level as control mice (Supplementary Figure 1D).

### FcγRs and C3 Influence Early Anti-fVIII Antibody Formation

**Figure 4:**
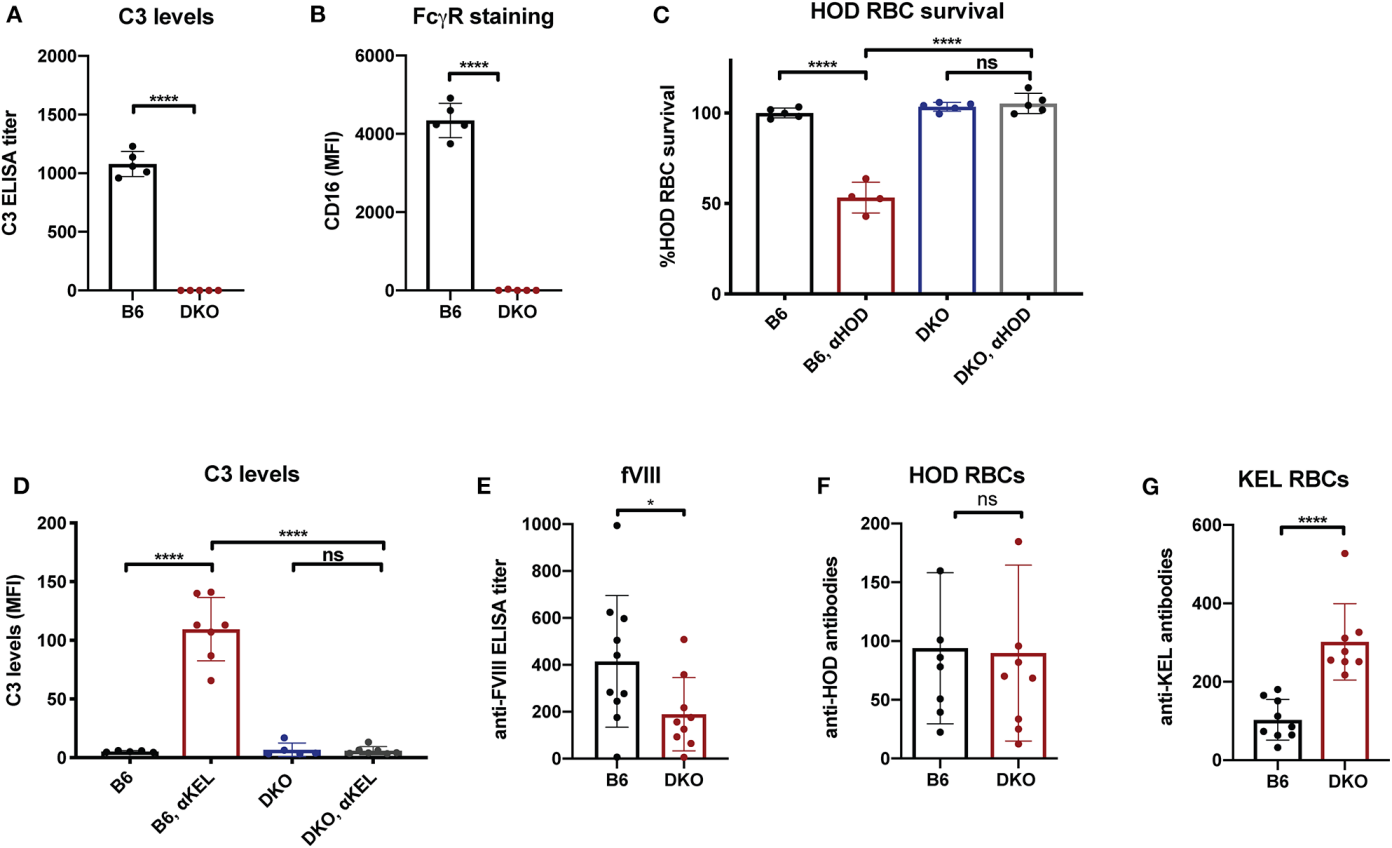
Mice deficient in both Fcγ receptors and C3 exhibit an impaired early antibody response to fVIII, but not to RBC alloantigens. **(A)** Analysis of C3 levels in WT or C3 X FcγR knockout recipients (DKO). **(B)** Quantiative analysis of Fcγ receptor levels in WT or C3 X FcγR knockout mice (DKO). **(C)** HOD (HEL, OVA, and Duffy) RBCs were transfused into non-immunized or anti-HOD (αHOD) immunized WT or C3 X FcγR knockout recipients, followed by evaluation for specific HOD RBC clearance. **(D)** KEL RBCs were transfused into non-immunized or anti-KEL (αKEL) immunized WT or C3 X FcγR knockout recipients (DKO), followed by examination for C3 deposition specifically on the KEL RBC surface. **(E)** WT or C3 X FcγR knockout recipients received three weekly injections of fVIII followed by evalution of anti-fVIII antibody formation by ELISA. **(F)** Flow cross match results obtained following serum incubation with HOD RBCs following transfusion of HOD RBCs into WT or C3 X FcγR knockout (DKO) recipients. **(G)** Flow cross match results obtained following serum incubation with KEL RBCs following transfusion of KEL RBCs into WT or C3 X FcγR knockout (DKO) recipients. ns = not significant. *p < 0.05, ****p < 0.0001.

While the immune responses to fVIII, HOD and KEL in the absence of FcγRs or C3 knockouts suggests that neither FcγRs nor C3 are individually required for antibody formation, whether FcγRs or C3 play a redundant role in the developing immune response to any of these antigens remains unknown. As antibodies can ligate FcγRs independent of complement and C3 activation could occur in FcγR knockouts, it remained possible that FcγRs or C3 may fill an important role in the developing immune response to fVIII when the other antibody effector system is absent. To control for potential redundancy between FcγRs and C3 in the developing immune response toward fVIII, we crossed FcγR and C3 knockouts to generate mice genetically deficient in both FcγRs and C3. To determine whether these double knockouts (FcγRs × C3 KOs) were deficient in FcγRs and C3, we first examined FcγRs on leukocyte surfaces and C3 in serum. Similar to FcγR and C3 knockouts individually, FcγRs × C3 KOs possessed no detectable C3 in their serum, nor could CD16 be detected on the leukocyte surface (Figures 4A,B). Furthermore, similar activity assays of incompatible transfusion employed previously likewise demonstrated that FcγRs × C3 KOs were devoid of functional FcγRs or C3 (Figures 4C,D).

Having confirmed that FcγRs × C3 KOs do not possess functional FcγRs and C3, we next sought to determine whether FcγRs and C3 are involved in anti-fVIII antibody formation. To accomplish this, FcγRs × C3 KO recipients were similarly injected with rfVIII, followed by evaluation of anti-fVIII antibody formation 21 days following the first infusion. Unlike the outcomes observed following fVIII injection into either FcγR or C3 KO recipients individually, FcγRs × C3 KO recipients generated an attenuated anti-fVIII antibody response when compared to similarly injected WT recipients (Figure 4E). To determine whether the ability of HOD or KEL RBCs to induce antibodies is also influenced by both FcγR and C3, HOD or KEL RBCs were transfused into FcγRs × C3 KO or WT recipients, followed by evalution of anti-HOD or ant-KEL antibody formation, respectively. Unlike fVIII, HOD RBCs were not only able to induce anti-HOD antibodies in FcγRs × C3 KO recipients, but anti-HOD antibody levels in these recipients were comparable to that observed in WT recipients transfused in parallel (Figure 4F). Similar to KEL RBC transfusion in C3 knockout recipients, KEL RBCs actually induced an increased anti-KEL antibody response in FcγRs × C3 KO recipients when compared to WT transfused in parallel (Figure 4G).

### Additional fVIII Injection Boosts Anti-fVIII Antibodies Independent of FcγRs and C3

**Figure 5:**
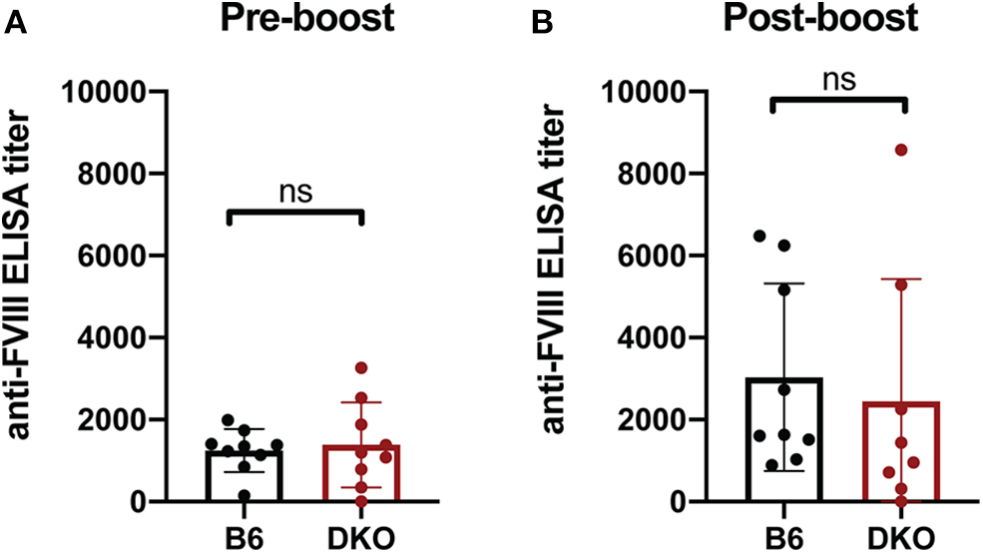
Increases in anti-fVIII antibody formation following additional fVIII exposure occurs independent of Fcγ receptors and C3. **(A)** WT or C3 X FcγR knockout recipients (DKO) received an intial three weekly injections of 2 μg fVIII followed by an additional 2 μg fVIII injection and evalution by ELISA of anti-fVIII antibody formation 4 weeks following initial fVIII exposure. **(B)** WT or C3 X FcγR knockout (DKO) recipients knockout recipients received an intial four weekly injections of 2 μg fVIII followed by an additional 4 μg fVIII injection and evalution of anti-fVIII antibody formation 6 weeks following initial fVIII exposure by ELISA. ns = not significant.

As FcγRs engage IgG antibodies and IgG antibodies also possess the ability to fix complement, it remains possible that the potential consequences of FcγRs and C3 on anti-fVIII antibody formation are not fully realized until after higher levels of anti-fVIII antibodies develop following initial rounds of fVIII exposure. As a result, we next infused previously anti-fVIII immunized FcγRs × C3 KO recipients with additional fVIII. Similar to the outcome observed following early formation of anti-fVIII antibodies in FcγR × C3 KOs, fVIII exposure at 4 weeks following initial fVIII exposure readily occurred in FcγR × C3 KO recipients (Figure 5A). Importantly, anti-fVIII antibodies were not only present in FcγR × C3 KOs at this time point, but the levels of antibodies failed to differ from WT recipients evaluated in parallel. To determine whether these existing anti-fVIII antibodies may impact a fVIII-induced boost of anti-fVIII antibody formation, we next injected additional fVIII into previously immunized FcγRs × C3 KO recipients and evaluated anti-fVIII antibody levels 2 weeks later. A boost dose of fVIII delivered in this manner resulted in similar levels of anti-fVIII antibody formation in FcγRs × C3 KO recipients as occurred WT mice (Figure 5B). Taken together, these results suggest that neither FcγRs or C3 are required for the formation of additional anti-fVIII antibodies once initial anti-fVIII antibody development occurs.

## Discussion

Anti-fVIII alloantibodies can develop in patients with hemophilia A following fVIII infusion, and may not only directly limit the therapeutic options for this patient population but can also increase morbidity and mortality (3–9). However, no prophylactic strategy currently exists that can actively prevent inhibitor formation. The inability to prevent inhibitor formation in at-risk patients in part stems from a fundamental lack of understanding regarding key pathways that initiate this process. In order to effectively understand risk factors that may predict the likelihood of inhibitor development and then prevent this process in at-risk patients, key factors that regulate the development of anti-fVIII alloantibodies must be identified.

Recent studies suggest that several early cellular mediators may facilitate the development of anti-fVIII antibody formation following fVIII exposure. Removal of the spleen can significantly attenuate fVIII antibody formation (20), suggesting that key constituents within the spleen may be important in the development of anti-fVIII antibodies. Consistent with this, depletion of marginal zone macrophages (MZM), other macrophage populations that reside in the MZ or marginal zone B cells can inhibit anti-fVIII antibody formation (20, 21). These results suggest that MZ B cells, MZM and perhaps other MZ constituents may work in concert to initiate fVIII inhibitor formation. Previous studies also demonstrate that MZM, in particular, and MZ B cells work in collaboration to trap and then respond to circulating foreign antigen (55–58). Following engagement of antigen by MZM and MZ B cells, MZ B cells possess the capacity to potently activate CD4 T cells (59, 60). In addition, MZ B cells can traffic antigen to B cell follicles (58, 61, 62), where they can actively facilitate CD4 T cell-dependent immune responses by delivering antigenic substrate to the germinal center (GC) reaction (59, 63). fVIII infusion increases T cell responses and enhances T follicular helper (TFH) cell numbers (22, 29), strongly suggesting that MZ B cells, MZM and TFH cells work in concert to drive anti-fVIII antibody formation. Importantly, marginal sinus constituents may not only be responsible for driving anti-fVIII antibody formation, as recent results suggest that these cells may also facilitate antibody formation following exposure to other antigens delivered intravascularly, including RBC transfusion (53).

While previous studies suggest a role for possible early immune cells that may initiate an anti-fVIII immune response, which immune factors drive these and perhaps other cells to generate antibodies in this setting has remained largely unknown. Similar to other alloantigens, fVIII possess no known adjuvant properties, but instead represents an otherwise innocuous antigen. Pre-existing, naturally occurring antibodies have been shown to recognize fVIII, suggesting that antibody engagement of fVIII following initial infusion may facilitate fVIII uptake and presentation to other immune cells, presumably through FcγRs (41, 42, 45). Antibodies that form in direct response to fVIII infusion would be predicted to also facilitate additional anti-fVIII antibody formation in a similar manner. FcγRs are expressed on numerous immune cells thought to participate in anti-fVIII antibody formation, including key macrophages and dendritic cell populations (64–67). Direct interactions between antibodies and fVIII would therefore be predicted to enhance fVIII uptake (64–67), as clear receptors capable of recognizing and facilitating fVIII uptake by antigen presenting cells (APC) remain to be fully defined. The ability of anti-fVIII antibodies to increase fVIII uptake by APCs *in vitro*, while also enhancing *de novo* anti-fVIII antibody formation *in vivo* appears to corroborate this notion and led to the present studies. However, our findings unexpectedly suggest that *de novo* anti-fVIII antibody development occurs independent of FcγRs. Possible differences between previous studies and the present findings may reflect the impact of affinity matured antibodies engaging fVIII, which may redirect or otherwise influence the ongoing immune response in ways not observed when early anti-fVIII antibodies undergo affinity maturation over time or when naturally occurring antibodies bind fVIII *in vivo*. Thus, while individual affinity matured anti-fVIII antibodies may influence fVIII uptake and immunogenicity, it is unclear whether polyclonal antibodies that develop in direct response to fVIII exposure similarly influence fVIII antibody formation during an ongoing immune response. However, it is certainly possible that anti-fVIII antibodies may induce immune complex formation that enhances fVIII removal and overall immune recognition completely independent of FcγRs. Future studies will be needed to examine these distinct possibilities.

Recent data suggest that in addition to FcγRs, C3 can regulate anti-fVIII antibody formation. These data are completely consistent with a large body of data demonstrating that C3 is required for, or strongly influences, productive antibody responses against a broad range of antigens (68–73). While C3 and its split products can impact a wide variety of immune cells, C3 engagement of B cells in particular is thought to directly enhance B cell activation and eventual differentiation into antibody secreting cells (72). MZ B cells are defined by high expression of complement receptor 1 (CR1 or CD21) (60), which is thought to sensitize these innate-like B cells to C3 decorated antibody-antigen complexes (69, 74, 75). Given the role of MZ B cells and other marginal zone constituents in the development of anti-fVIII antibodies and recent results demonstrating that complement depletion with cobra venom factor (CVF) can negatively impact fVIII immunization (27), we fully expected an attenuated or even absent response to fVIII following injection into C3 knockout recipients. Indeed, given the apparent bimodal response to fVIII following CVF treatment (27), these previous results were consistent with the possibility that incomplete depletion or recrudescence of C3 following CVF injection may sustain some level of anti-fVIII antibody formation; variable pharmacological responses to CVF between animals could have reflected these differences. However, the development of anti-fVIII antibodies appeared to occur unabated in C3 knockout mice. Confirmatory studies, which included ELISA analysis of C3 antigen levels and *in vivo* C3 activity assays using a well-defined model ruled out the distinct possibility that residual C3 levels may be present and therefore contribute to the ongoing anti-fVIII immune response in these animals. Administration of nCVF to hemophilia A and WT mice prior to fVIII exposure failed to significantly alter very early anti-fVIII antibody formation compared to controls, although effective complement depletion could certainly impact later anti-fVIII antibody formation in this setting. Taken together, these results suggest that at least in some settings and at certain time points in the evolution of the immune response, C3-independent pathways of anti-fVIII antibody formation may exist.

Differences in C3 removal between distinct methods of CVF-induced depletion vs. genetic deletion, or variances in a number of environmental factors, including microflora or other stimuli, may in part account for differences observed in anti-fVIII antibody formation between prior studies and the present data. CVF has been used for decades to explore complement biology *in vivo* and therefore represents a valid and commonly used tool to define the role of complement in a variety of settings (76, 77). CVF injection depletes complement by first activating several key elements of the complement cascade (78), which through a consumptive process, ultimately results in complement elimination. In contrast, C3 knockout recipients are deficient in C3 from birth. While CVF can certainly deplete complement, initial CVF-mediated complement activation can result in the rapid release of complement split products, which can be very potent immune modulators (79–83). To avoid anti-CVF antibody interference when using nCVF in the present study, we examined anti-fVIII antibody formation 2 weeks after injection when prior nCVF injections still effectively deplete complement (84). However, using this approach, we unexpectedly failed to observe a difference in anti-fVIII antibody formation at this early time point. These data do not demonstrate that CVF fails to impact anti-fVIII antibody formation, as prior studies examined antibody development at later time points where CVF may influence anti-fVIII antibody formation (27); inherent limitations in our model of CVF injection precluded us from being able to directly test this possibility. While the timing of antibody evaluation is most likely responsible for differences in study outcomes, it is possible that differences in nCVF and humanized CVF could also influence observations. Unlike humanized CVF, the nCVF utilized in the current study leads to the generation of C5a that has been shown to modulate antigen presenting cells (85, 86), which may further affect the immune response to fVIII. Differences in the kinetics and magnitude of complement split product formation following nCVF or humanized CVF injection, such as iC3b and C3d, could also result in distinct outcomes as these complement products have also been shown to influence immune responses (27, 48, 87, 88). Further exploring these possibilities may provide novel approaches to inducing tolerance or at least inhibiting the immune response to fVIII. Another, perhaps more subtle, possibility is the influence of housing conditions on immune responses to otherwise innocuous antigens. Unlike infectious challenge, induction of antibodies to fVIII occurs in the complete absence of known adjuvant and therefore may be more sensitive to subtle differences in environmental conditions, such as the microflora composition. As these types of environmental stimuli have been shown to influence immune responses in other settings (89, 90), such differences may also impact the relative contribution of complement in anti-fVIII antibody formation. Although directly testing this possibility would certainly be challenging, exploring the potential influence of microbiota on the role of complement in regulating early anti-fVIII formation in future studies may provide insight into this possibility.

While it is not known whether robust complement activation or its early consequences influences anti-fVIII antibody formation, recent studies demonstrated that vaccination at the time of fVIII administration can actually diminish anti-fVIII antibody development (91). These results raise the possibility that certain immune activators may induce immune deviation from an optimal baseline state needed to effectively induce antibodies against fVIII following exposure. However, not all immune activators are the same. Recent results suggest that poly I:C, a viral-like mimetic, can significantly enhance anti-fVIII antibody formation, in addition to other antigens delivered intravascularly (21, 51, 92). These results illustrate that a variety of factors, some of which may be environmental in nature, may influence subtle immune outcomes, especially following exposure to otherwise innocuous antigens such as fVIII.

In contrast to the outcome of fVIII injection into individual FcγR knockout or C3 knockout recipients, exposure of recipients deficient in both FcγRs and C3 resulted in an attenuated early immune response to fVIII. The reduced response observed in FcγRs × C3 KOs following fVIII injection suggests that both of these antibody effector systems may play a role in early fVIII immune recognition. Multiple immune cells possess FcγRs and complement receptors, raising the possibility that either C3 or antibody engagement may enhance fVIII uptake and removal (65, 67, 93). As many distinct cell populations can express various FcγRs and complement receptors, how these receptor systems may work in concert to facilitate an early immune response to fVIII remains unknown. One possibility is that FcγRs or complement receptors facilitate fVIII recognition and removal, which may result in the activation of APCs, alter cytokine secretion, enhance migration of neighboring cells, facilitate antigen presentation to cognate T cells or some combination of the above. While *in vitro* assays can begin to dissect some of the key players that may be involved in such a pathway, recent studies suggest that T cell activation *in vitro* to innocuous antigens may not recapitulate actual APC-mediated activation *in vivo* (94). Although several early immune players, such as MZ B cells and MZM, have been identified as key regulators of anti-fVIII antibody formation (20, 21), APC populations and additional downstream regulators responsible for anti-fVIII antibody development *in vivo* remain incompletely defined. As each APC population can expresss distinct FcγRs or complement receptors (95, 96), the relative engagement of each antibody effector system is possibly dicated by the APC predominately responsible for T cell activation *in vivo*. As a result, identifying the key cell or cells responsible for these downstream events will greatly facilitate efforts to define how FcγRs or complement influence anti-fVIII antibody formation. Furthermore, while initial anti-fVIII antibody formation appeared to be influenced by FcγRs and C3 effector systems, antibody formation was not absent in FcγRs × C3 KOs, suggesting that a variety of cells and receptors may contribute to early anti-fVIII antibody formation. The combined influence of FcγRs and C3 appears to further support this possibility, suggesting redundant and potentially complementary roles in these antibody effectors and perhaps other systems capable of facilitating early fVIII recognition and response.

In contrast to fVIII, HOD RBCs induced similar anti-HOD antibody levels in FcγRs × C3 KOs, while KEL RBCs actually induced an increased anti-KEL response in FcγRs × C3 KOs when compared to WT recipients. Like the development of inhibitors, alloantibodies against RBC alloantigens can cause significant complications in patients (50, 97–100). However, despite similarities in the clinical challenges these alloantibodies can create, the results of the present study suggest that distinct features of alloantigens may influence the relative impact of the different immune pathways they engage and suggest that each antigen may induce alloantibodies through distinct immune pathways. Despite the unique ways in which FcγRs or C3 can influence immune responses to fVIII, KEL or HOD, there are features of the immune response to these antigens that do appear to bear some similarities. Similar to fVIII, transfused RBCs localize to the marginal sinus and depletion of MZ B cells also prevents antibody responses following RBC transfusion (53). The immune responses to fVIII and RBC alloantigens bear other similarities clinically. While individuals chronically exposed to fVIII or RBC antigens can experience alloantibody formation, not all patients respond, suggesting that additional factors may influence responder status (8, 100). As the disease state and the genetic backgrounds of patients with hemophilia or transfusion-dependent conditions can fundamentally differ, these clinical observations suggest that other factors may influence the likelihood that individuals respond. Previous studies suggest that polymorphisms in FcγRs may influence the likelihood of RBC alloimmunization or vaccination responses (101, 102). Although a similar examination of anti-fVIII antibody formation has yet to be reported, the data presented here suggest that complete absence of FcγRs does not influence antibody formation against fVIII or RBC antigens. However, polymorphisms that enhance antibody interactions with FcγRs, which were not tested in the present model, could influence this process. While less is known regarding the potential impact of C3 in the development of anti-RBC antibodies clinically, these results also suggest that antibody formation in these settings can occur independent of C3. In contrast, C3 appears to attenuate anti-KEL antibody formation, possibly by influencing the involvement of CD4 T cells (48). It should be noted, however, that different levels of C3 activation may influence the likelihood of anti-fVIII antibody formation and that this approach may therefore serve as a useful tool to redirect baseline immune function in such a way as to reduce or even prevent anti-fVIII antibody development.

As with any study, limitations should be considered. It is important to acknowledge that FcγRs knockout, C3 knockout, and FcγRs X C3 KO mice have normal levels of murine fVIII. This stands in stark contrast to prior studies examining the immune response to fVIII using hemophilia A mice and may account for some differences observed between the present and prior studies. While recent studies have likewise examined anti-fVIII immune responses in WT mice (29), as noted, prior studies have primarily examined the potential role of various immune players in the development of anti-fVIII antibodies in mice completely deficient or partially deficient in fVIII (20, 21, 26–28, 43). Similar to the present study, examination of immune response toward RBC antigens has taken an analogous approach, wherein WT mice or mice genetically deficient in particular immune factors are used as recipients of RBC transfusion, but are not genetically deficient in the target antigen, such as KEL or Duffy. These mice instead express the mouse version of the human blood group antigen transgenically expressed on mouse RBCs (50, 103, 104). However, even in this setting, similar to fVIII injection, recipients generate robust immune responses to these antigens, presumably against portions of the antigen not shared by the mouse protein. Recent results demonstrate that fVIII remains immunogenic even after removing its hemostatic activity (39), suggesting that fVIII's role in hemostasis is not required for its ability to serve as an antigenic substrate. fVIII injections can also induce anti-fVIII antibodies in the absence of von Willebrand factor (vWF) (39), suggesting that fVIII does not need to displace endogenous fVIII in WT recipients to induce an immune response. While prior studies have induced anti-fVIII antibodies following injection of human fVIII into mice (20, 21, 26–28, 43), which technically results in xenoantigen exposure, whether a similar immune response occurs following exposure to murine fVIII represents an important outstanding question. However, despite studies suggesting fVIII activity or engagement of vWF may not play a critical role in the development of anti-fVIII antibodies, the potential impact of endogenous fVIII expression on the immune response to exogenous, antigenically distinct fVIII remains unknown and certainly deserves additional examination. Such differences could account for distinct observations following injection of fVIII into WT or hemophilia A mice; exploring the potential influence of endogenous fVIII on the immune response to exogenous fVIII, especially where differences in the potential influence of key immune regulators such as complement may have been observed, certainly deserves additional attention in future studies.

While the use of a hemophilia A model when examining anti-fVIII antibody formation certainly has advantages, coupling hemophilia A mice with commonly employed knockout strategies to define key players in this process can be challenging. Indeed, while knockout approaches represent the most common and robust strategies to study fundamental aspects of immunology in model systems, the significant time required to cross mice to generate double and triple knockout animals is often time and cost prohibitive. In addition, alteration of gene function in murine knockout models may promote the development of compensatory mechanisms, leading to changes in expression of additional genes that may ultimately affect the overall immune response to fVIII. While elucidating any potential secondary effects, and determining which cell population or popluations may be responsible is beyond the scope of the current study, they should be considered when interpreting the results. However, while these challenges in studying anti-fVIII antibody formation remain, examination of anti-fVIII antibody formation in fVIII sufficient mice certainly has inherent limitations as noted above. Despite these limitations, the overall observations presented here could provide important insight and raise fundamental questions regarding antibody formation against fVIII that may be relevant for future studies.

Taken together, the differential ability of fVIII and other RBC antigens to induce immune responses in FcγR, C3, and FcγR × C3 KOs suggests that the immune response to these antigens fundamentally differ from each other and also from more commonly studied antigens often employed to study basic principles of immunology. Not only do these antigens fail to possess known features capable of activating immunity, they also do not appear to be universally influenced by common factors thought to drive or at least facilitate antibody formation following exposure to other antigens. These results, therefore, not only provide unique insight into immune pathways involved in anti-fVIII and anti-RBC antibody formation, but suggest that the immune pathways engaged by these clinically relevant antigens may fundamentally differ from previously studied antigens.

## Data Availability Statement

All datasets generated for this study are included in the article/Supplementary Material.

## Ethics Statement

All experiments were performed under animal protocols approved by the Institutional Animal Care and Use Committee of Emory University.

## Author Contributions

PZ, CA, SM, and SSt conceived of the project, which was facilitated by CM, AM, SSh, JA, SC, WB, CC, HV, RJ, and CT who provided critical reagents, experimental support, and critical discussion. PZ and SSt wrote the manuscript, which was additionally commented on and edited by the remaining authors.

## Conflict of Interest

The authors declare that the research was conducted in the absence of any commercial or financial relationships that could be construed as a potential conflict of interest.

## Supplementary Material

Supplementary Figure 1 Early anti-fVIII antibody formation occurs independent of C3 depletion in WT and hemophilia A mice. **(A)** nCVF was administered to WT (B6) and hemophilia A mice followed by evaluation of plasma C3 levels at baseline and 6, 12, and 24 h after nCVF injection. **(B)** WT or hemophilia A mice underwent two weekly injections of 7.5 U nCVF (red arrows) followed by a 2 μg fVIII injection (black arrows) 6 h later. Plasma was collected (blue arrows) 24 h after nCVF administration for evaluation of C3 levels and 1 week following the second fVIII administration for evaluation of anti-fVIII antibodies. **(C)** Plasma C3 levels in WT and hemophilia A mice measured 24 h after saline (black) or nCVF (red) administration after the first (CVF #1) and second (CVF #2) weekly CVF injections. **(D)** Evaluation of anti-fVIII antibody formation in WT and hemophilia A mice 1 week after the second weekly dose of fVIII with saline (black) or nCVF (red). ns = not significant. ^****^*p* < 0.0001.
