# Supplementary figures and images for: Fc Gamma Receptors and Complement Component 3 Facilitate Anti-fVIII Antibody Formation
Source: Front Immunol. 2020 Jun 9;11:905. doi: 10.3389/fimmu.2020.00905 (PMC7295897; doi:10.3389/fimmu.2020.00905)

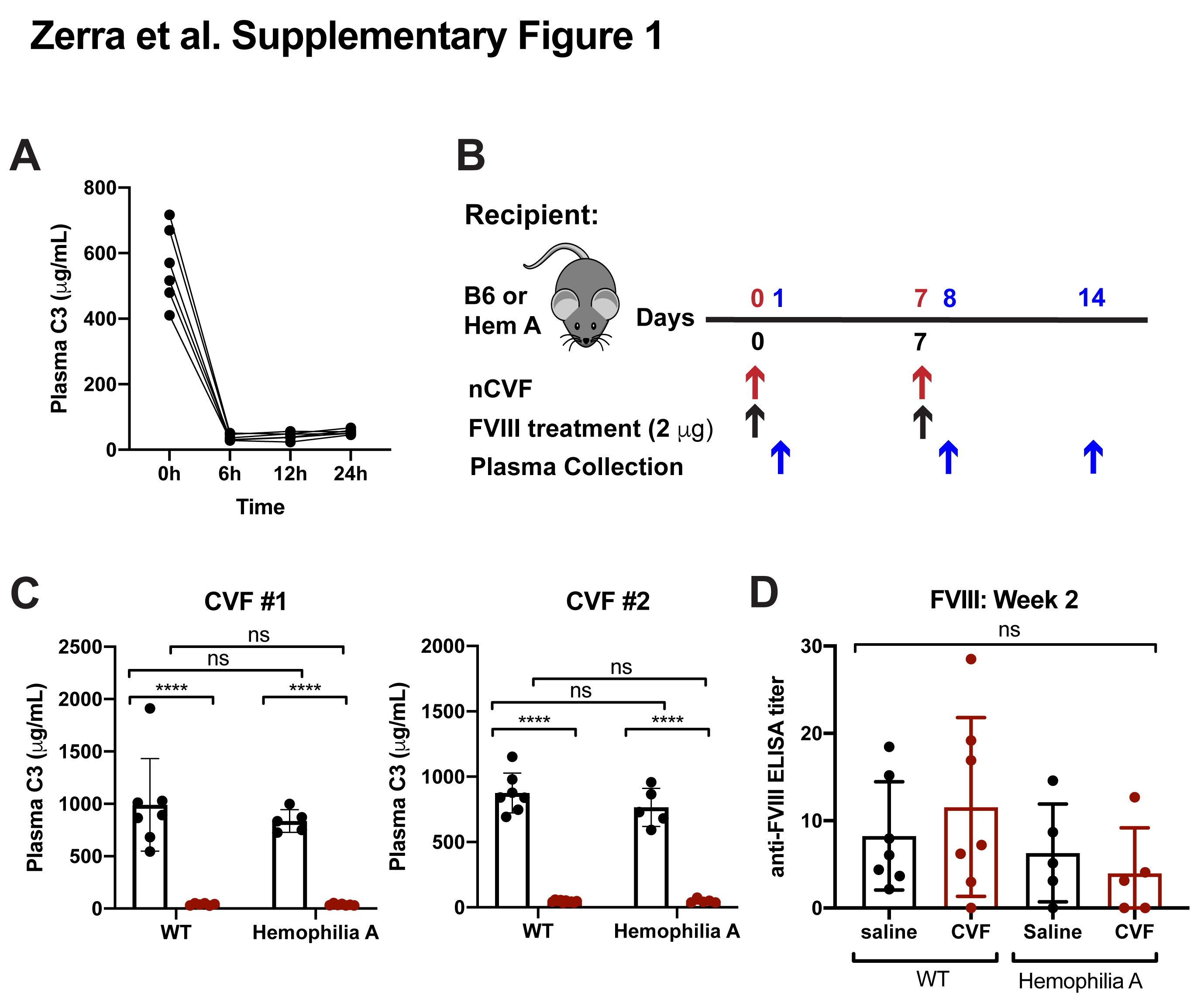

Supplement: Supplementary Figure 1 — Early anti-fVIII antibody formation occurs independent of C3 depletion in WT and hemophilia A mice. (A) nCVF was administered to WT (B6) and hemophilia A mice followed by evaluation of plasma C3 levels at baseline and 6, 12, and 24 h after nCVF injection. (B) WT or hemophilia A mice underwent two weekly injections of 7.5 U nCVF (red arrows) followed by a 2 μg fVIII injection (black arrows) 6 h later. Plasma was collected (blue arrows) 24 h after nCVF administration for evaluation of C3 levels and 1 week following the second fVIII administration for evaluation of anti-fVIII antibodies. (C) Plasma C3 levels in WT and hemophilia A mice measured 24 h after saline (black) or nCVF (red) administration after the first (CVF #1) and second (CVF #2) weekly CVF injections. (D) Evaluation of anti-fVIII antibody formation in WT and hemophilia A mice 1 week after the second weekly dose of fVIII with saline (black) or nCVF (red). ns = not significant. ****p < 0.0001. [file Image_1.tif]
